# Rough sets and Laplacian score based cost-sensitive feature selection

**DOI:** 10.1371/journal.pone.0197564

**Published:** 2018-06-18

**Authors:** Shenglong Yu, Hong Zhao

**Affiliations:** 1 Fujian Key Laboratory of Granular Computing and Application (Minnan Normal University), Zhangzhou, Fujian, China; 2 Key Laboratory of Data Science and Intelligence Application, Fujian Province University, Zhangzhou, Fujian, China; Tianjin University, CHINA

## Abstract

Cost-sensitive feature selection learning is an important preprocessing step in machine learning and data mining. Recently, most existing cost-sensitive feature selection algorithms are heuristic algorithms, which evaluate the importance of each feature individually and select features one by one. Obviously, these algorithms do not consider the relationship among features. In this paper, we propose a new algorithm for minimal cost feature selection called the rough sets and Laplacian score based cost-sensitive feature selection. The importance of each feature is evaluated by both rough sets and Laplacian score. Compared with heuristic algorithms, the proposed algorithm takes into consideration the relationship among features with locality preservation of Laplacian score. We select a feature subset with maximal feature importance and minimal cost when cost is undertaken in parallel, where the cost is given by three different distributions to simulate different applications. Different from existing cost-sensitive feature selection algorithms, our algorithm simultaneously selects out a predetermined number of “good” features. Extensive experimental results show that the approach is efficient and able to effectively obtain the minimum cost subset. In addition, the results of our method are more promising than the results of other cost-sensitive feature selection algorithms.

## Introduction

Feature selection [1–4] is an essential process for machine learning applications [5–7], because it improves generalization capabilities and reduces running time [8–10]. The goal of the feature selection problem is to find a feature subset to reduce the dimensionality of the feature space and improve the predictive accuracy of a classification algorithm [11–16]. There are various feature evaluation methods such as maximal margin [17], maximal stability [18], effective distance [19], maximum relevance-maximum significance [20], and matrix factorization subspace learning [21, 22]. These evaluation methods assume that the obtained data are free. However, in many real-world applications, we should pay test costs for collecting data items [23–25]. Test costs are often measured by time, money, and other resources [26]. Therefore, the cost must be considered in the feature selection process.

Cost-sensitive feature selection (CSFS) [27–29] focuses on selecting a feature subset with the minimal cost as well as one that preserves a particular property of the decision system [30–32]. The CSFS problem becomes a feature selection problem when the cost of CSFS problem is zero. Thus, the CSFS problem is more generalization than the feature selection problem, and it has attracted a lot of research interest recently. The main aim of CSFS algorithms is to search for the cheapest feature subset that preserves sufficient information for classification and clustering (see, e.g., [31, 33, 34]).

In recent years, there have been many work on cost-sensitive feature selection. Tan proposed cost-sensitive feature selection and used it in robotics [35]. Zhang proposed a cost-sensitive feature selection with respect to waiting cost [36]. Min used basic concepts of rough set theory to propose cost-sensitive attribute reduction [31]. Cost-sensitive feature selection based on decision-theoretic was proposed by Jia [37]. Zhao considered numerical data with measurement errors and proposed a cost-sensitive feature selection algorithm based on backtracking approach [38]. Yang proposed a backtracking algorithm for granular structure selection with minimal test cost in [39]. The Semi-greedy heuristics for cost-sensitive feature selection was proposed by Min in [40]. However, these heuristic algorithms evaluate each feature individually and select features one by one. They do not consider the relationship among features and have high time complexity.

In this paper, we propose a cost-sensitive feature selection algorithm based on Rough sets and Laplacian score (CSFS-RSLS) to address the CSFS by considering the trade-off between feature score and test cost. We aim to select a feature subset with maximal feature importance and minimal cost. Thus, each feature is evaluated by both Laplacian score and test cost. Laplacian score can evaluate features according to their locality preserving ability. The cost is given by three different distributions [31, 41] to simulate different applications. It is distinguished from the existing heuristic algorithms, the proposed algorithm takes into account the relationship among features and simultaneously selects out a predetermined number of “good” features.

Nine open datasets from the University of California-Irvine (UCI) library are employed to study the performance of our algorithm. The proposed algorithm is implemented in our open resource software called COst-SEnsitive Rough sets (COSER) [42]. The experimental results show that CSFS-RSLS can select an optimal feature subset with the best exponential weight setting. Compared with two heuristic algorithms [43, 44], CSFS-RSLS algorithm provides an efficient solution on eight datasets. In addition, our algorithm significantly reduces the time complexity and resource consumption.

In subsequent sections, we firstly presents the test-cost-sensitive decision system. Secondly, the subsection includes two points, one is the Laplacian score with cost sensitive, the other is our proposed algorithm. The CSFS problem in our algorithm is defined in this subsection. Subsequently, we show two evaluation metrics, which can evaluate the performance of our proposed CSFS-RSLS algorithm. Fourthly, we discuss the experiment process and list some settings and results. Finally, we provide conclusion and future work.

## Test-cost-sensitive decision system

In real applications, we consider decision system with test cost. The test-cost-sensitive decision system is a fundamental concept in data mining and machine learning. The first part shows the test-cost-sensitive decision system models. There are a number of measurement methods with different test costs to obtain a numerical data item. We define test-cost-sensitive decision system with error ranges in the second part.

**Definition 1** [45]. A test-cost-sensitive decision system (TCS-DS) *S* is the 6-tuple:

$$\begin{array}{c} S=(U,C,D,\left\{V_{a}|a\in C\cup D\right\},\left\{I_{a}|a\in C\cup D\right\},c^{*}), \end{array}$$ (1)

where *U* is a finite set of objects called the universal, *C* is the set of conditional features, *D* is the set of decision features. For each *a* ∈ *C* ∪ *D*, *I*_*a*_: *U* → *V*_*a*_. *V*_*a*_ is the set of values for each *a* ∈ *C* ∪ *D*, and *I*_*a*_ is an information function for each feature *a* ∈ *C* ∪ *D*, $c^{*}:2^{C}\to R^{+}\cup \left\{0\right\}$ is the feature subset test cost function, where $R^{+}$ is the set of positive real numbers.

We assume that the test sequence does not influence the total cost, and for each *A* ⊆ *C*, one should specify the value of c*(*A*). Therefore, the feature subset test cost function c* is to employ a vector

$$\begin{array}{c} c^{*}=\left[c^{*}\left(\emptyset \right),c^{*}\left(\left\{a_{1}\right\}\right),c^{*}\left(\left\{a_{2}\right\}\right),\cdots ,c^{*}\left(\left\{a_{1},a_{2}\right\}\right),\cdots ,c^{*}\left(C\right)\right]. \end{array}$$ (2)

The space requirement for storing function c* is 2^|*C*|^, which soon becomes unacceptable as |*C*| increases. To deal with this problem, we need to develop an alternative representation of the test cost function. Therefore, we set $c:C\to R^{+}\cup \left\{0\right\}$ is the test cost function and

$$\begin{array}{c} c^{*}\left(A\right)=\sum_{a\in A}c^{*}\left(\left\{a\right\}\right)=\sum_{a\in A}c\left(a\right), \end{array}$$ (3)

we assume that the range of cost function *c* is non-negative $\left(R^{+}\cup \left\{0\right\}\right)$, which is a natural assumption in reality. A feature test cost function can easily be represented by a vector *c* = [*c*(*a*_1_), *c*(*a*_2_), ⋯, *c*(*a*_|*C*|_)].

**Definition 2** [43]. Let *S* = (*U*, *C*, *D*, *V*, *I*, c*, *e*) be a TCS-DS-ER, where *U*, *C*, *D*, *V*, *I* and c* have the same meaning as Definition 1, $e:C\to R^{+}\cup \left\{0\right\}$ is the maximal error range of *a* ∈ *C*, and ±*e*(*a*) is the error range of *a*. The error range of feature *a* is defined as

$$\begin{array}{c} e\left(a\right)=\Delta \frac{\sum_{i=1}^{m}a\left(x_{i}\right)}{m}, \end{array}$$ (4)

where we set Δ = 0.1, *a*(*x*_*i*_) is the *i*-th instance value of *a* ∈ *C*, *i* ∈ [1, *m*], and *m* is the number of instances. The precision of *e*(*a*) can be adjusted through Δ setting.

In order to facilitate processing and comparison, the conditional feature values are normalized, and their value range from 0 to 1. In fact, there are a number of normalization approaches. We employ a simple function of normalization: *y* = (*x* − *min*)/(*max* − *min*), where *y* is the normalized value, *x* is the initial value, and *max* and *min* are the maximal and minimal values in each conditional features.

Table 1 presents a decision system of *Bupa liver disorder* (*Liver* for short), which conditional features are normalized values; where *U* = {*x*_1_, *x*_2_, ⋯, *x*_345_}, *C* = {Mcv, Alkphos, Sgpt, Sgot, Gammagt, Drinks}, and *D* = {Selector}. Table 2 presents an example of test cost vector.

**Table 1:** A numerical decision system (*Liver*).

Patient	Mcv	Alkphos	Sgpt	Sgot	Gammagt	Selector
*x*_1_	0.53	0.60	0.27	0.29	0.09	1
*x*_2_	0.53	0.36	0.36	0.35	0.06	2
*x*_3_	0.55	0.27	0.19	0.14	0.17	2
*x*_4_	0.68	0.48	0.20	0.25	0.11	2
*x*_5_	0.58	0.41	0.05	0.30	0.02	2
*x*_6_	0.87	0.28	0.06	0.16	0.04	2
*x*_7_	0.61	0.34	0.11	0.16	0.01	2
⋯	⋯	⋯	⋯	⋯	⋯	⋯
*x*_344_	0.68	0.39	0.15	0.27	0.03	1
*x*_345_	0.87	0.66	0.35	0.52	0.21	1

**Table 2:** An example of test cost vector.

*a*	Mcv	Alkphos	Sgpt	Sgot	Gammagt
*c*(*a*)	$16.00	$20.00	$45.00	$28.00	$33.00

## Rough sets and Laplacian score based cost-sensitive feature selection

In this section, we introduce the relative reduct by Rough sets, the Laplacian score (LS) and our algorithm. The first part describes the relative reduct in numeric data. The second part describes the use of LS in cost-sensitive feature selection. Our CSFS-RSLS algorithm is described in the last part. The key of the exponential weighting algorithm is the feature importance exponent weighted function, test costs, and a user-specified exponent *α*.

### Relative reducts in rough sets

Rough set theory [46], proposed in the early 1980s, is a mathematical tool to deal with uncertainty and is a relatively new soft computing method. Concept of relative reduct has been thoroughly investigated by the rough set theory. The concept of relative reduct is built on decision systems, and there are many different definitions, such as positive approximation reducts [47], parallel reducts [48, 49], and a general definition reducts [50].

**Definition 3**. Any *B* ⊆ *C* is a decision-relative reduct if *POS*_*B*_(*D*) = *POS*_*C*_(*D*), and ∀*a* ∈ *B*, *POS*_*B*−{*a*}_(*D*) ⊂ *POS*_*B*_(*D*).

The first condition guarantees that the information in terms of the positive region is preserved, and the second condition guarantees that no superfluous test is included. With this decision-relative reduct, decision-relative core is naturally defined as follows.

**Definition 4**. Let *Red*(*S*) denotes the set of all decision-relative reducts of *S*. The decision-relative core of *S* is *Core*(*S*) = ∩*Red*(*S*).

In other words, *Core*(*S*) contains those tests appearing in all decision-relative reducts. A decision-relative reduct is also called a reduct for brevity.

### Laplacian score in cost-sensitive feature selection

In real-world applications, the LS can be applied to supervised or unsupervised feature selection. For many datasets, the local structure of the space is more important than the global structure. To represent the local geometry of the data, LS is used to construct a nearest-neighbor graph. The nearest-neighbor graph of the LS is based on the observation that, two data points are probably related to the same topic if they are close to each other. This can be well-preserved under the data structure and the nearest neighbor graph can be obtained. The importance of each feature is calculated from the nearest neighbor graph. For each feature, the basic idea of LS is to evaluate the feature importance according to its locality preserving power. Here, we apply the LS to unsupervised feature selection.

For each feature, we assume that the feature importance is *LS*(*a*), *a* ∈ *C*. We combine feature importance and cost as follows:

$$\begin{array}{c} LS\left(a,c\right)=LS\left(a\right)c{\left(a\right)}^{\alpha }, \end{array}$$ (5)

where *α* is a user-specified non-positive exponent and *c* is the cost of feature. If *α* = 0, this function reduces to the traditional feature importance. About function *LS*(*a*) [51], let’s set some symbols. Let *LS*(*a*_*r*_) denote the Laplacian Score of the *r*-th feature. Let *f*_*ri*_ denote the *i*-th sample of the *r*-th feature, *i* = 1, ⋯, *m*. Our function can be stated as the following four main steps:

1. Construct a nearest neighbor graph *G* with *m* samples. The *i*-th sample corresponds to *x*_*i*_. We put an edge between samples *i* and *j* if *x*_*i*_ and *x*_*j*_ are “close”, i.e. *x*_*i*_ is among *k* nearest neighbors of *x*_*j*_ or *x*_*j*_ is among *k* nearest neighbors of *x*_*i*_. When the label information is available, one can put an edge between two nodes sharing the same label.
2. If samples *i* and *j* are connected, put $S_{ij}=e^{-\frac{\|x_{i}-x_{j}{\|}^{2}}{t}}$, where *t* is a suitable constant. Otherwise, put *S*_*ij*_ = 0. The weight matrix *S* of the graph models the local structure of the data space.
3. For the *r*-th feature, we define **f**_*r*_ = [*f*_*r*1_, *f*_*r*2_, ⋯, *f*_*rm*_]^*T*^, *D* = *diag*(*S***1**), **1** = [1, ⋯, 1], and *L* = *D* − *S*, where the maxtrix *L* is often called graph Laplacian. Let
  $$\begin{array}{c} {\tilde{f}}_{r}=f_{r}-\frac{f_{r}^{T}D1}{1^{T}D1}1. \end{array}$$ (6)
4. Compute the LS of the *r*-th feature as follow:
  $$\begin{array}{c} LS\left(a_{r}\right)=\frac{{\tilde{f}}_{r}^{T}L\tilde{f}}{{\tilde{f}}_{r}^{T}D\tilde{f}}. \end{array}$$ (7)

**Example 1** Firstly, we use a subtable of Table 1 as shown in Table 3 and obtain an error range vector in Table 4 by Table 3. Secondly, we obtain the core feature is Gammagt by Table 4 and set *k* = 3 and *t* = 1, the weight matrix *S* can be written as follow:

$$\begin{array}{c} S=(\begin{array}{cccccccccc} 1 & & 0 & & 0 & & 0.81 & & 0.80\; & 0\\ 0 & & 1 & & 0.77 & & 0.86 & & 0\; & 0\\ 0 & & 0.77 & & 1 & & 0.76 & & 0\; & 0\\ 0.81 & & 0.86 & & 0 & & 1 & & 0\; & 0\\ 0.80 & & 0 & & 0 & & 0.81 & & 1\; & 0\\ 0 & & 0.75 & & 0 & & 0.73 & & 0\; & 1 \end{array}). \end{array}$$

**Table 3:** A subtable of the *Liver* decision system.

Patient	Mcv	Alkphos	Sgpt	Sgot	Gammagt	Selector
*x*_1_	0.53	0.60	0.27	0.29	0.09	1
*x*_2_	0.68	0.36	0.20	0.35	0.12	2
*x*_3_	0.55	0.27	0.19	0.14	0.17	2
*x*_4_	0.68	0.48	0.20	0.25	0.11	1
*x*_5_	0.58	0.57	0.05	0.30	0.08	2
*x*_6_	0.87	0.28	0.06	0.20	0.09	1

**Table 4:** An error range vector.

*a*	Mcv	Alkphos	Sgpt	Sgot	Gammagt
*e*(*a*)	0.06	0.04	0.02	0.03	0.01

Then, for each feature, the Laplacian Score *LS*(*a*) is shown in Table 5.

**Table 5:** A feature importance vector of the *Liver* subtable.

*a*	Mcv	Alkphos	Sgpt	Sgot	Gammagt
*LS*(*a*)	0.8456	0.7439	0.9506	0.9345	0.9680

The value of the *LS*(*a*) indicates the quality of the feature. Table 5 shows that the feature importance is **Gammagt** > **Sgpt** > **Sgot** > **Mcv** > **Alkphos**. When we add the cost and set *α* = −1, the *LS*(*Mcv*, *c*) = *LS*(*Mcv*)*c*(*Mcv*)^−1^ = 0.8456 × 16^−1^ = 0.052; *LS*(*Alkphos*, *c*) = 0.037; *LS*(*Sgpt*, *c*) = 0.021; *LS*(*Sgot*, *c*) = 0.033; *LS*(*Gammagt*, *c*) = 0.029; Obviously, after considering the feature importance, the cost and the core. We choose the order is: **Gammagt** > **Mcv** > **Alkphos** > **Sgot** > **Sgpt**. As opposed to considering only feature importance or cost, the result is very different.

### The proposed algorithm

To more quickly and efficiently deal with the problem of test cost, we propose a feature-importance function that includes cost sensitivity to calculate the feature score. This function combines feature importance and cost, and is more reasonable and more widely applicable to practical problems. The algorithm pseudocode is listed in Algorithm 1 and contains two main steps:

1. Add the core feature to *B* according to reduct of rough sets;
2. Add the current-best feature to *B* according to feature importance function *LS*(*a*, *c*) until the number of *B* set achieve the desired number of features.

**Algorithm 1** Rough sets and Laplacian score based cost-sensitive feature selection (CSFS-RSLS).

**Input**: (*U*, *C*, *D*, {*V*_*a*_|*a* ∈ *C* ∪ *D*}, {*I*_*a*_|*a* ∈ *C* ∪ *D*}, *c*)

**Output**: A feature subset with minimal test cost

**Method**: CSFS-RSLS

1:  *B* = ∅;

  //Core computing

2:  **for** (*i* = 1; *i* ≤ |*C*|; *i* + +)**do**

3:   **if** (*POS*_*C* − {*a*_*i*_}_(*D*) ≠ *POS*_*C*_(*D*))**then**

4:    *B* ← *B* ∪ {*a*_*i*_}; //{*a*_*i*_} is a core feature

5:   **end if**

6:  **end for**

7:  For any *a* ∈ *C*, compute *LS*(*a*, *c*); //compute the laplacian score for each feature

  //Addition feature

8:  *CA* = *C* − *B*;

9:  Select *d* features *a* ∈ *CA* with the maximal *LS*(*a*, *c*);

10:  *B* ∪ {*a*};

11:  return *B*;

Lines 7 and 9 are key to this algorithm. In line 7, we can insert different feature importance significance functions *LS*(*a*, *c*) to obtain various algorithms. Line 9, where *d* is determined by the comparison algorithm, selects the feature subset number. Eq (4) is introduced here to adjust the influence of the test cost. We use a comparison method in the proposed algorithm to choose the best features subset for different *α* values.

Algorithm 1 has the following three main advantages over existing algorithms:

1. Computation time is reduced. Because the time complexity of the backtracking algorithm for finding a dataset reduction is 2^∣*C*∣^, where |*C*| is the feature number of the dataset, when |*C*| is large, the calculation time is impractically high. Algorithm 1 shows that the time complexity of the CSFS-RSLS algorithm is |*C*|.
2. It can handle large datasets, which a number of existing algorithms cannot handle. Large datasets have a very large number of features, and existing algorithms would find it very difficult to operate on them. However, in our algorithm, because the time complexity of the CSFS-RSLS algorithm is |*C*|, the CSFS-RSLS algorithm is suitable for large datasets.
3. It is highly likely to produce feature subsets with the minimal total cost. For instance, in Table 2, the existing algorithm obtains the feature subset {Mac, Alkphos, Gammagt}, which has a total test cost is $16 + $20 + $33 = $69. Our algorithm obtains the feature subset fMac, Alkphos, Sgotg, and its total test cost is $16 + $20 + $28 = $64. Obviously, our algorithm is better.

## Evaluation method

Among the existing algorithms, there are many algorithms to deal with the MTR problem. It is necessary to define several evaluation methods to compare the performances. First, we need a method to evaluate the quality of one feature subset. For example, if the test cost of the optimal feature subset is $100, an equal number of feature subsets with test cost $120 are better than another with a test cost of $150. We propose algorithm can run on many datasets or one dataset with different test cost settings. We propose two statistical metrics: average below factor and average exceeding factor.

### Below factor

For a dataset produce test cost setting, let *R*′ be an optimal reduct. The below factor of a feature subset *R* is

$$\begin{array}{c} bf\left(R\right)=\frac{c^{*}\left(R^{\prime }\right)-c^{*}\left(R\right)}{c^{*}\left(R^{\prime }\right)}. \end{array}$$ (8)

The below factor is a quantitative metric for evaluating the performance of a feature subset. It shows the goodness of a feature subset when it is better than the optimal. Naturally, if *R* is an optimal feature subset, the below factor is 0.

#### Maximal below factor

To demonstrate the performance of the algorithm, statistical metrics are needed. Let the number of experiments be *K*. In the *i*-th experiment (1 ≤ *i* ≤ *K*), the feature subset computed by the algorithm is denoted by *R*_*i*_. The maximal below factor (MBF) is defined as

$$\begin{array}{c} \max_{1\leq i\leq K}bf\left(R_{i}\right). \end{array}$$ (9)

This is the best case of the algorithm given the dataset. To some extent, it can express the performance of this algorithm.

#### Average below factor

The average below factor (ABF) is defined as

$$\begin{array}{c} \frac{\sum_{i=1}^{K_{1}}bf\left(R_{i}\right)}{K_{1}}. \end{array}$$ (10)

Because ABF is averaged over *K*_1_ different test cost settings, the value of *K*_1_ is c*(*R*) less than c*(*R*′). This value is a very good way to show the performance of the algorithm from a solely statistical perspective.

### Exceeding factor

For a dataset with a test cost setting, the exceeding factor is used to show the performance of the algorithm. Similarly, if the algorithm is run *K* times, the exceeding factor and the maximal exceeding factor are defined in [31]. The exceeding factor provides a quantitative metric to evaluate the performance of a feature subset. It shows the badness of a feature subset when it is not optimal. The value of the maximal exceeding factor is the worst case for some datasets. Although it relates to the performance of one particular feature subset, it should be viewed as a statistical rather than individual metric.

The average exceeding factor (AEF) is defined as

$$\begin{array}{c} \frac{\sum_{i=1}^{K_{2}}ef\left(R_{i}\right)}{K_{2}}. \end{array}$$ (11)

The maximal exceeding factor is averaged on *K*_2_ = *K* − *K*_1_ different test cost settings. It is a statistical metric that represents the overall performance of the algorithm.

## Experiments

In this section, we try to answer the following questions by experimentation.

1. Is the running time of our algorithm reduced?
2. Is our algorithm efficient?
3. Is the CSFS-RSLS algorithm effective?
4. Is our algorithm appropriate for the minimal test cost feature selection problem?
5. Is there an optimal setting of *α* that is valid for any dataset?

### Datasets

Nine standard datasets are used to study the efficiency and effectiveness of the proposed CSFS-RSLS algorithm. The nine standard datasets of Machine Learning Databases are: *Liver*, *Wpbc*, *Promoters*, *Voting*, *Ionosphere*, *Credit*, *Prostate-GE*, *SMK-CAN-187*, and *Waveform*. The *SMK-CAN-187* [52] is a benchmark microarray based gene expression database and it has 187 samples and 19993 features. The other 8 datasets are from the UCI [53] library. Where *Liver* and *Wpbc* datasets are from medical applications. The *Promoters* dataset is from game applications, *Voting* dataset is from society applications, *Ionosphere* dataset is from physics applications, and *Credit* dataset is from commerce applications. *Prostate-GE* dataset has 102 samples and 5966 features from medical applications, the *Waveform* dataset has 5000 samples and 40 features from Vocality applications.

The data of our experiments come from real applications. However, because these datasets do not provide the test cost, we use uniform, normal, and pareto distributions to generate random test costs in [1, 100]. To help show the performance of the cost-sensitive feature selection algorithm, we create these data for the experiments. The data underlying this study have been uploaded to Github and are accessible using the following link: https://github.com/fhqxa/PLOSONE-D-17-34607.

Their basic information are listed in Table 6, where |*C*| is the number of features, and |*U*| is the number of instances, |*D*| is the number of classes.

**Table 6:** Datasets information.

No.	Name	Domain	|*U*|	|*C*|	|*D*|
1	Liver	Clinic	345	6	2
2	Wpbc	Clinic	198	33	2
3	Promoters	Game	106	57	2
4	Voting	Society	435	16	2
5	Ionosphere	Physics	351	34	2
6	Credit-g	Commerce	1000	20	2
7	Waveform	Vocality	5000	40	3
8	Prostate-GE	Clinic	102	5966	2
9	SMK-CAN-187	Society	187	19993	2

### Comparison of three distributions

For each dataset, we have different *α* values, and there are three distributions for generating the test cost settings. The algorithm is run 100 times with different test cost settings and different *α* settings on nine datasets.

**Fig 1:**
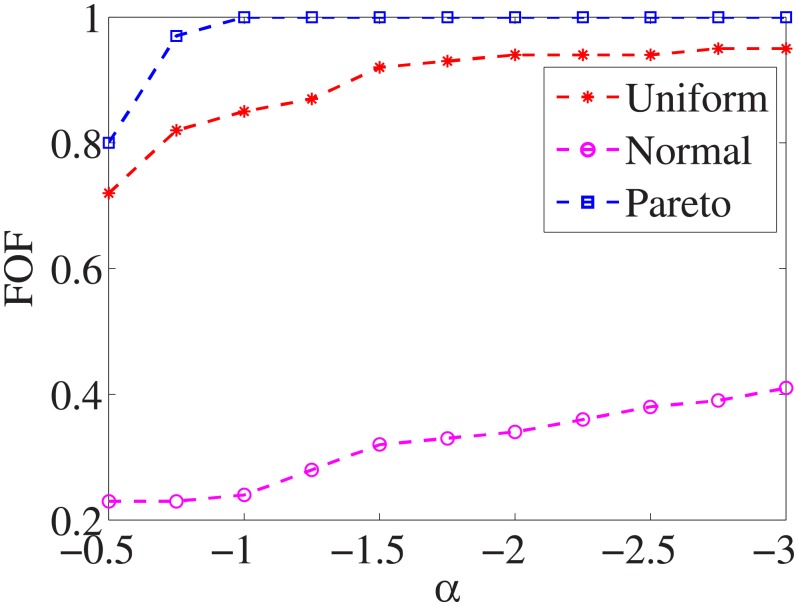
Finding optimal factor of Liver dataset.

**Fig 2:**
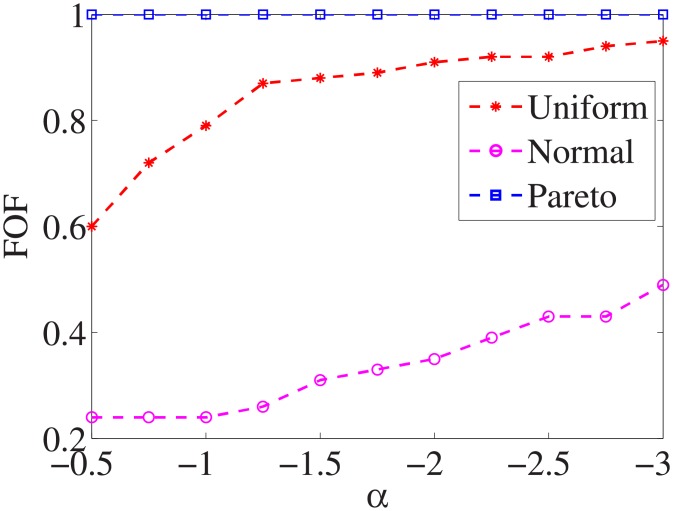
Finding optimal factor of Wpbc dataset.

**Fig 3:**
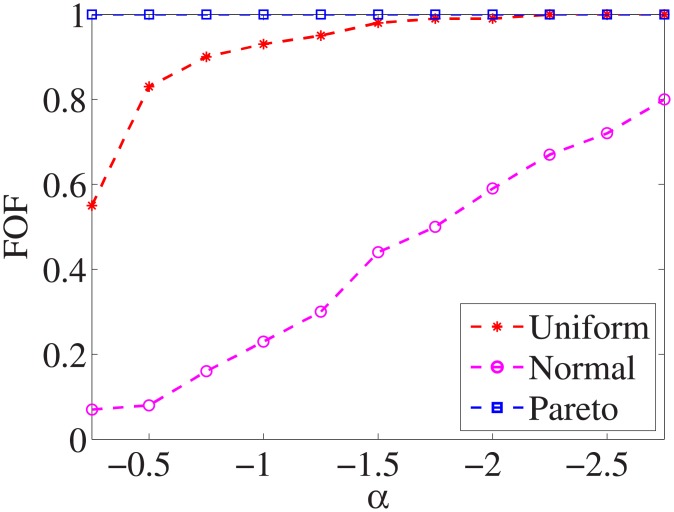
Finding optimal factor of Promoters dataset.

**Fig 4:**
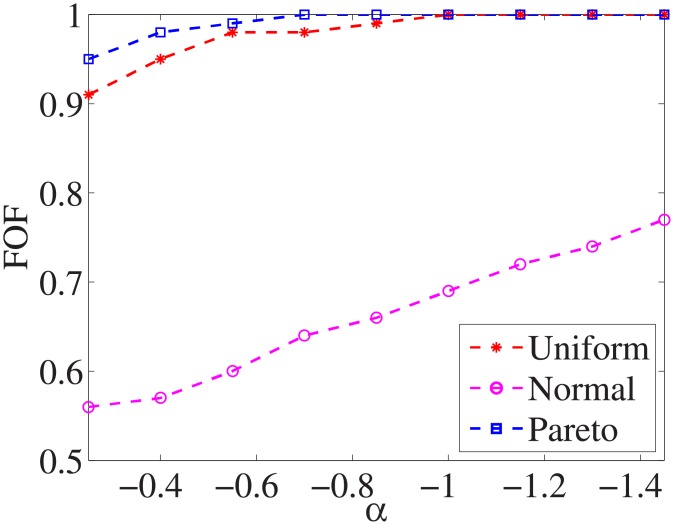
Finding optimal factor of Voting dataset.

**Fig 5:**
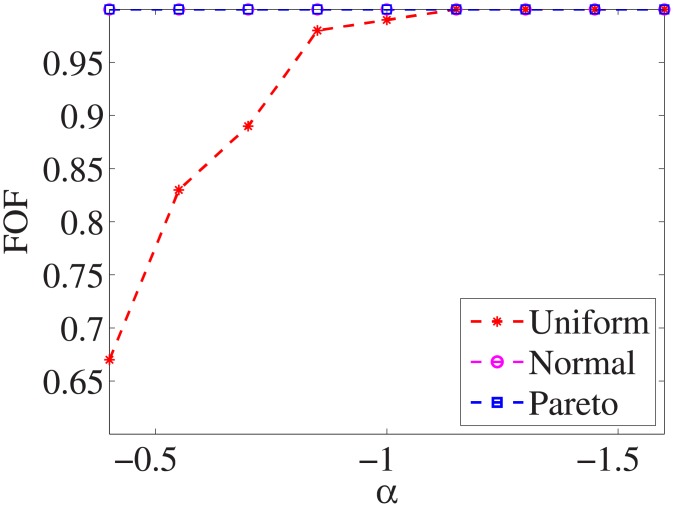
Finding optimal factor of Ionosphere dataset.

**Fig 6:**
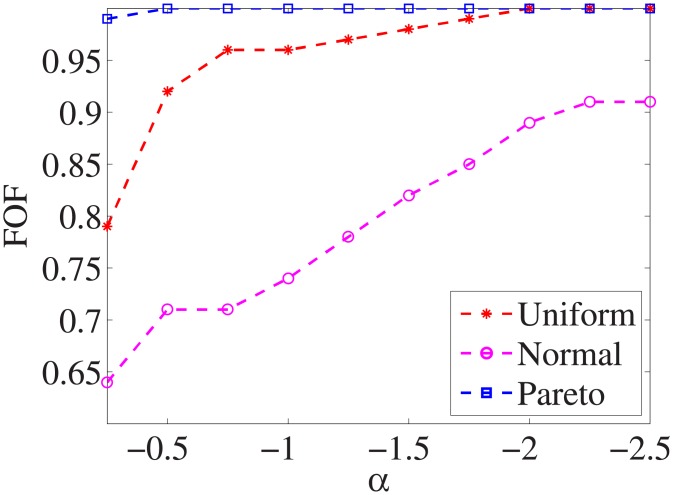
Finding optimal factor of Credit-g dataset.

**Fig 7:**
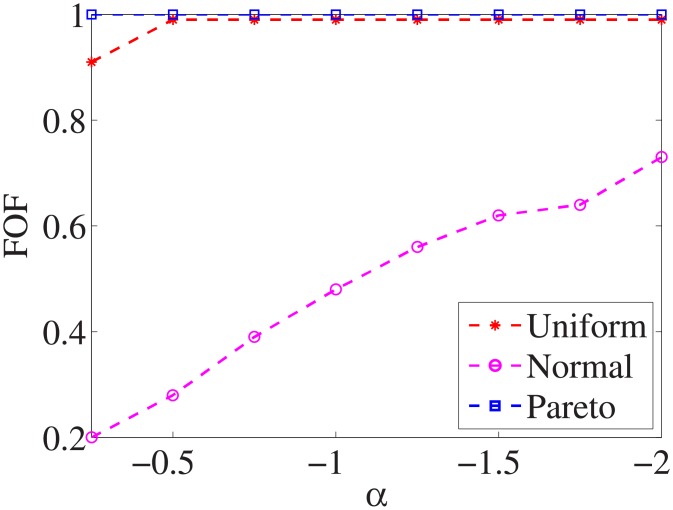
Finding optimal factor of Prostate-GE dataset.

**Fig 8:**
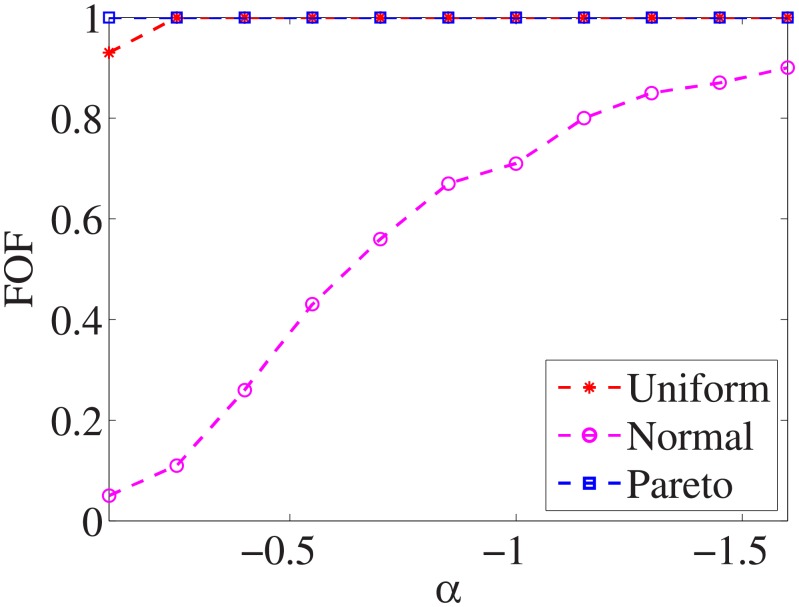
Finding optimal factor of SMK-CAN-187 dataset.

**Fig 9:**
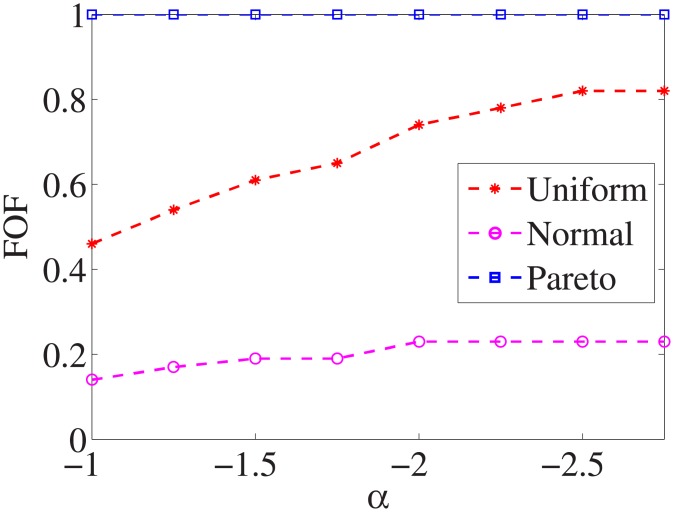
Finding optimal factor of Waveform dataset.

Figs 1–9 show the results of finding the optimal factors from the three distributions. The proposed algorithm performs the best with the pareto distribution for each dataset. Except for the *Ionosphere* dataset, the normal distribution leads to the worst performance. A possible reason is that the pareto distribution generates many small values and a few large values, and there are many features with both low test costs and large LSs. In contrast, the normal distribution generates many values close to the mean value, and there are no low test costs and large LSs. Finally, in the uniform distribution, there are more cheap tests than in the normal distribution, and fewer cheap tests than in the pareto distribution.

**Fig 10:**
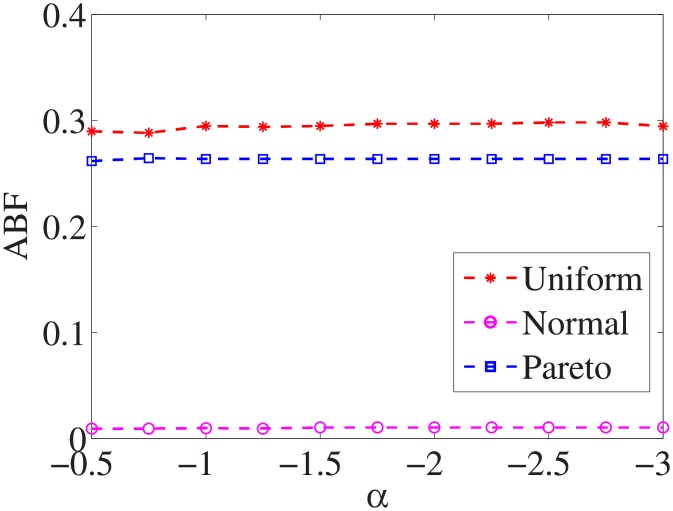
Average below factor of Liver dataset.

**Fig 11:**
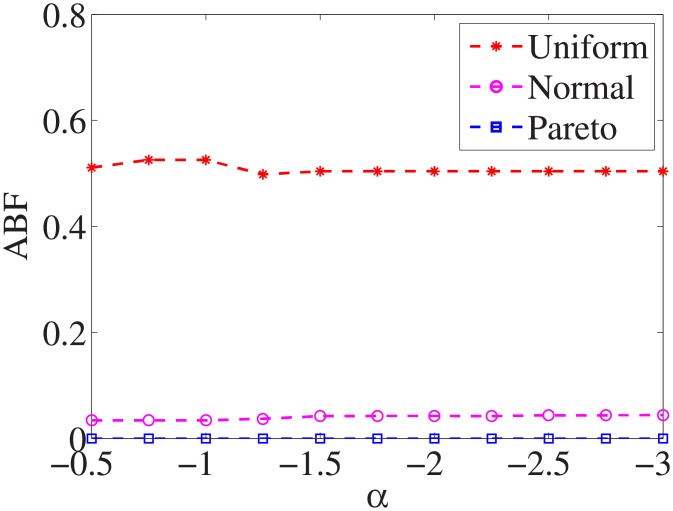
Average below factor of Wpbc dataset.

**Fig 12:**
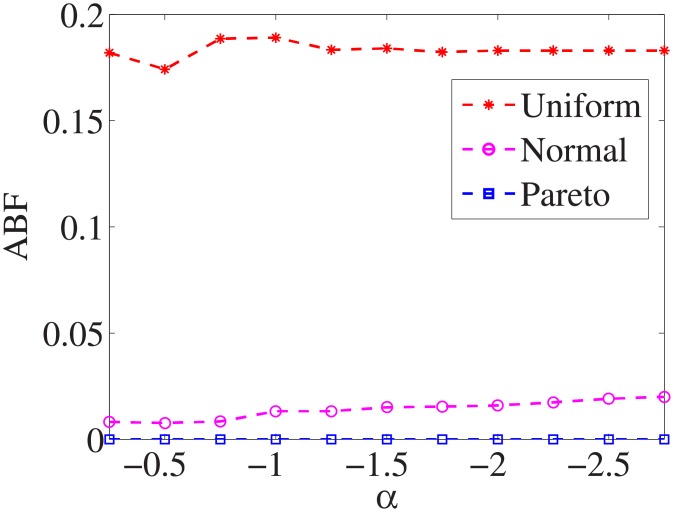
Average below factor of Promoters dataset.

**Fig 13:**
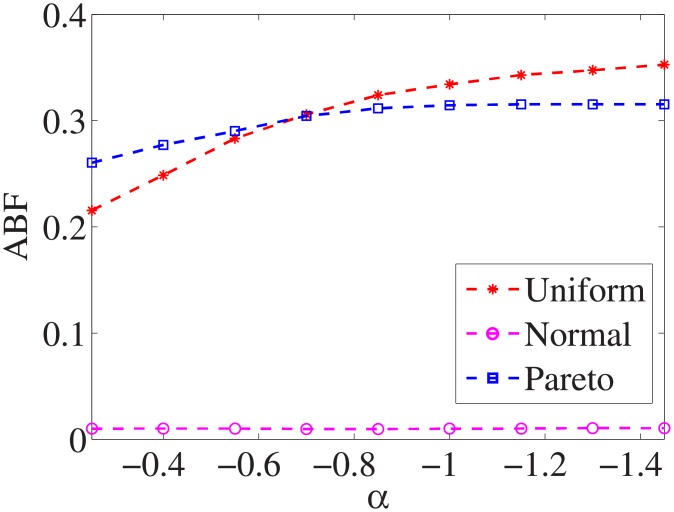
Average below factor of Voting dataset.

**Fig 14:**
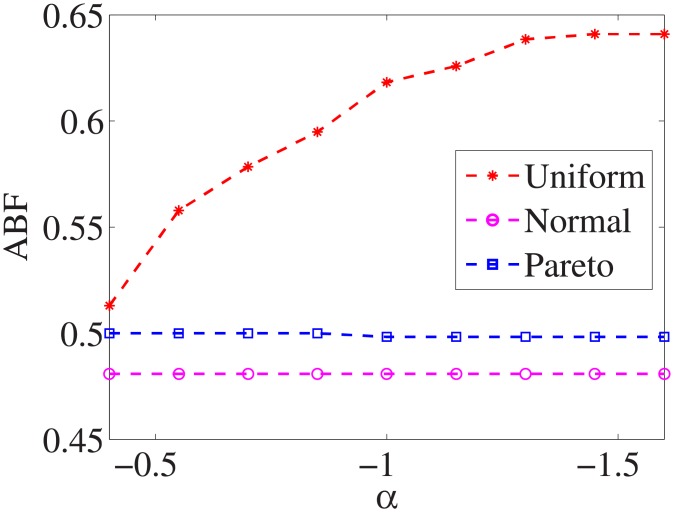
Average below factor of Ionosphere dataset.

**Fig 15:**
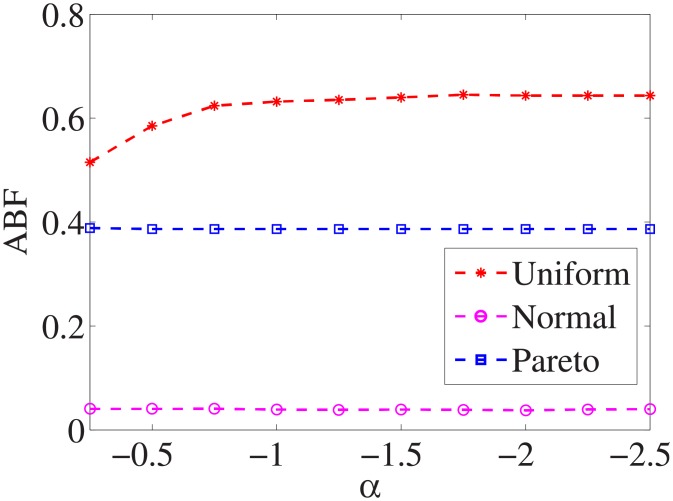
Average below factor of Credit-g dataset.

**Fig 16:**
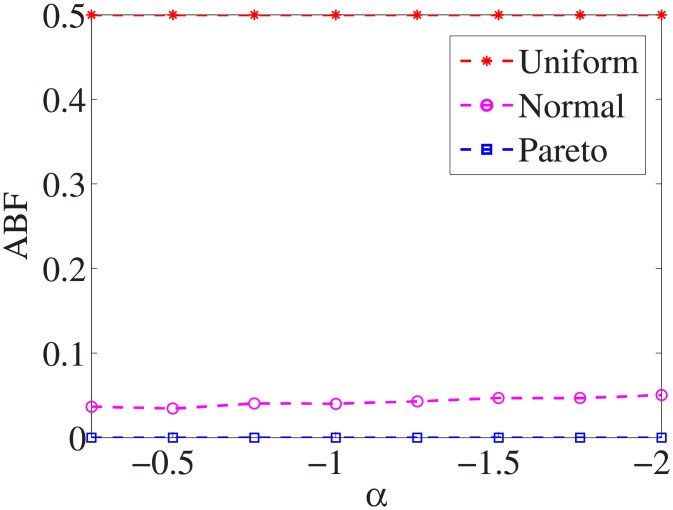
Average below factor of Prostate-GE dataset.

**Fig 17:**
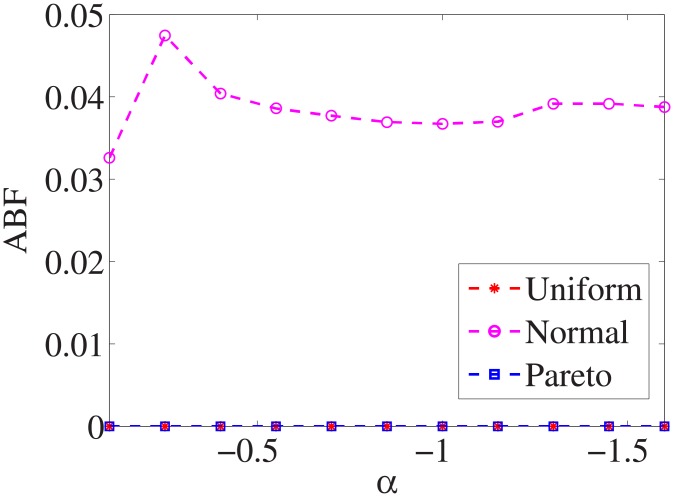
Average below factor of SMK-CAN-187 dataset.

**Fig 18:**
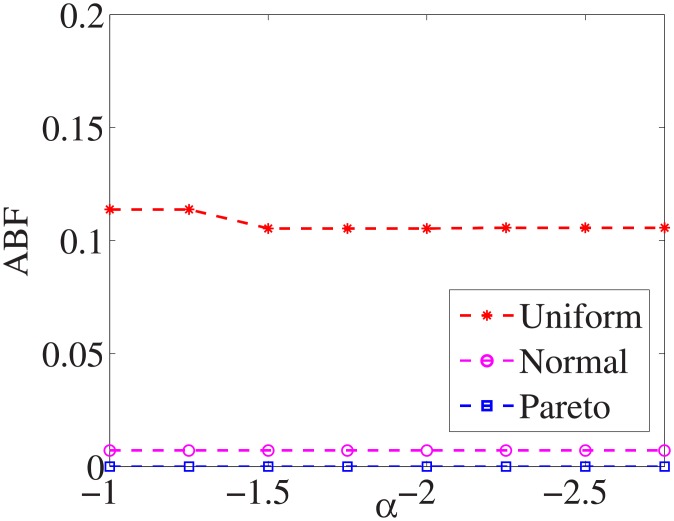
Average below factor of Waveform dataset.

Figs 10–18 show the average below factor. For the three distributions, the average below factor is more convincing than the maximal below factor because the average below factor is created from a statistical point of view. Hence, for the three distributions, the average below factor can better describe the performance of the CSFS-RSLS algorithm. From these results, we can see that the proposed algorithm obtains the best performance for each dataset from the uniform distribution except for the *SMK-CAN-187* dataset. With the pareto distribution, the average factor is 0 for the *Wpbc*, *Promoters*, *Prostate-GE*, *SMK-CAN-187*, and *Waveform* datasets. These results indicate that the test cost of the feature subset and the optimal reduction is the same. In the *Ionosphere* dataset, although the optimal factor is 1, the average below factor is not 0 but about 0.5. This result shows that the test cost of the feature selection subset is less than the test cost of the optimal reduction and is half that of the optimal reduction.

**Fig 19:**
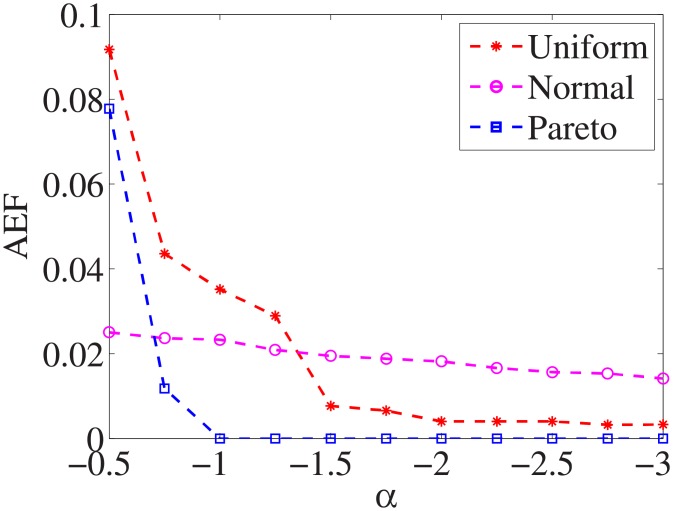
Average exceeding factor of Liver dataset.

**Fig 20:**
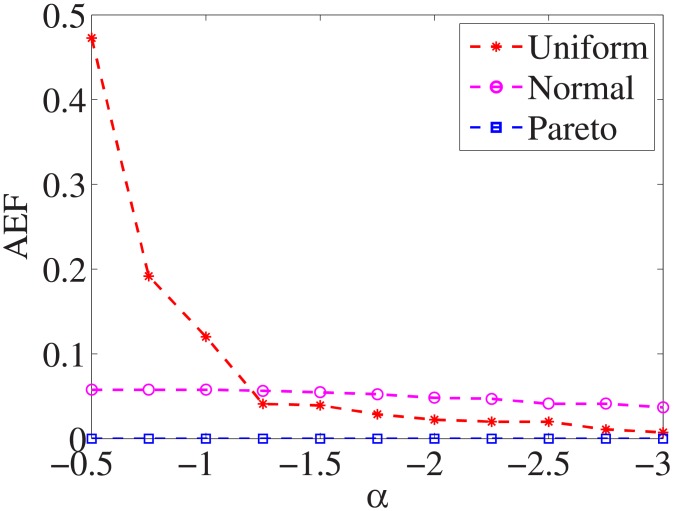
Average exceeding factor of Wpbc dataset.

**Fig 21:**
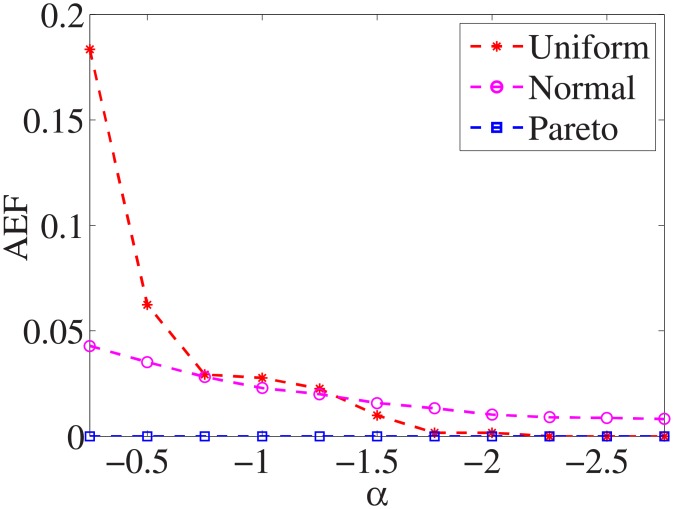
Average exceeding factor of Promoters dataset.

**Fig 22:**
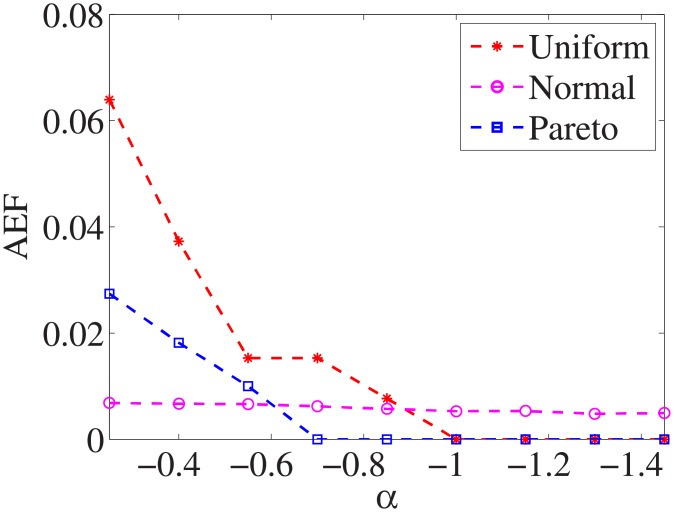
Average exceeding factor of Voting dataset.

**Fig 23:**
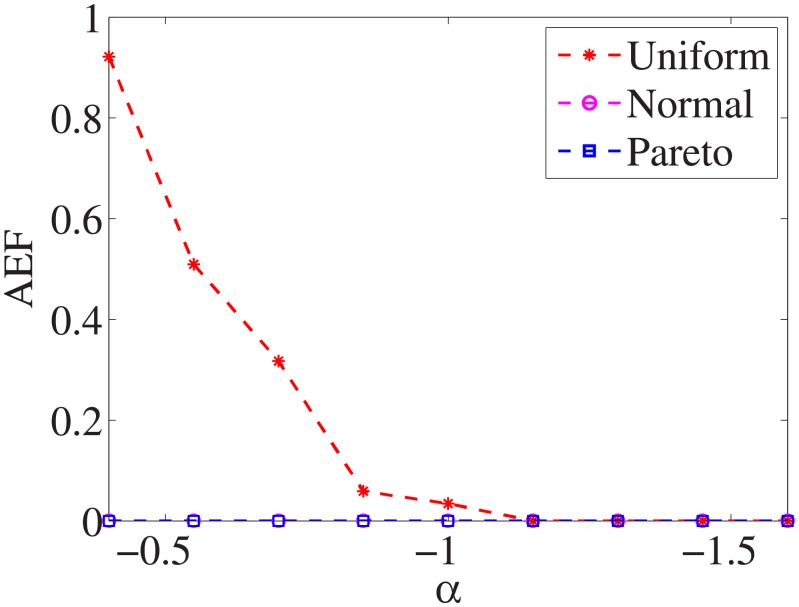
Average exceeding factor of Ionosphere dataset.

**Fig 24:**
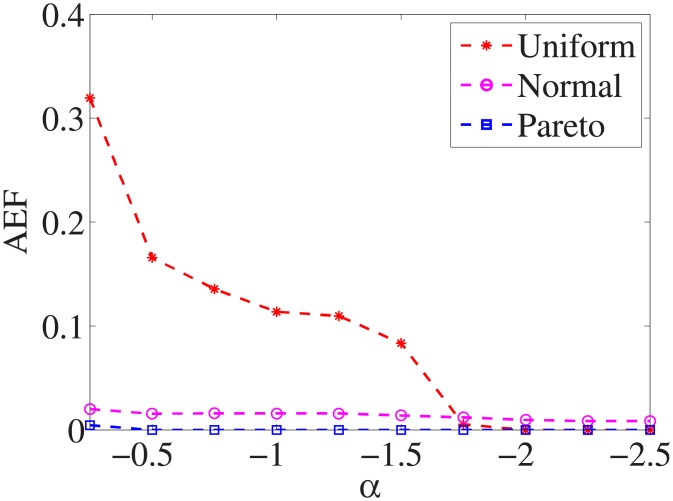
Average exceeding factor of Credit-g dataset.

**Fig 25:**
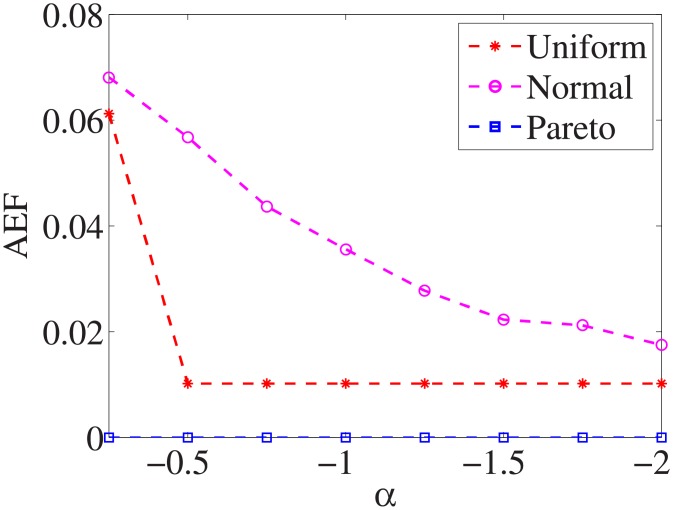
Average exceeding factor of Prostate-GE dataset.

**Fig 26:**
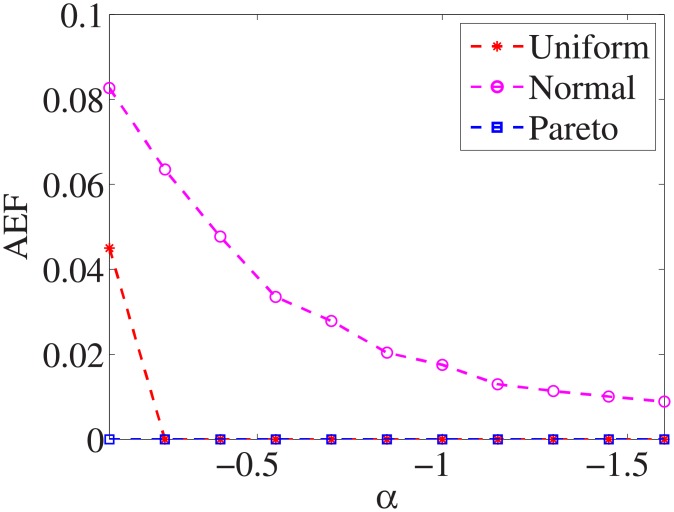
Average exceeding factor of SMK-CAN-187 dataset.

**Fig 27:**
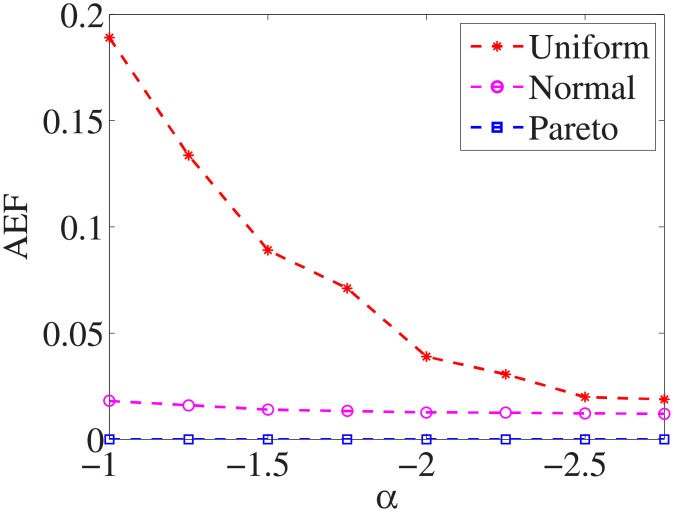
Average exceeding factor of Waveform dataset.

Figs 19–27 show the average exceeding factors. Here, the optimal setting for *α* is very close, if not equal, to that for the finding optimal factor. We only need to obtain the optimal setting to find the optimal factor. When this *α* is at the optimal setting, the average exceeding factor is very low. For example, it is 0 for the nine datasets with the pareto distribution. That is, the constructed feature subsets do not have a higher test cost than that of the optimal reduction, on average. This performance would very satisfactory for practical applications.

In Table 7, we list the results of each dataset to compare the two approaches according to the optimal factor. Both methods are based on CSFS-RSLS. The first approach, called the non-weighting approach, is implemented by setting *α* = 0. The second approach, called the average *α* approach.

**Table 7:** Results for *α* = 0 and *α* with the optimal setting.

Dataset	*α* = 0			optimal *α*		
	Uniform	Normal	Pareto	Uniform	Normal	Pareto
Liver	0.145	0.220	0.443	0.894	0.319	0.979
Wpbc	0.018	0.235	0.703	0.854	0.337	1.000
Promoters	0.000	0.040	0.295	0.920	0.415	1.000
Voting	0.440	0.510	0.523	0.979	0.661	0.991
Ionosphere	0.086	1.000	0.898	0.929	1.000	1.000
Credit-g	0.220	0.553	0.548	0.957	0.796	0.999
Prostate-GE	0.003	0.126	0.716	0.980	0.488	1.000
SMK-CAN-187	0.003	0.018	0.741	0.994	0.565	1.000
Waveform	0.000	0.000	0.438	0.678	0.201	1.000

We observe the following:

1. The non-weighting approach only performs well on the *Ionosphere* dataset relative to the other datasets. The non-weighting approach has the highest average value of 0.661 on the *Ionosphere* dataset. For the eight other datasets, the results are unacceptable. For the *Waveform* dataset, when *α* = 0, it obtains an optimal factor of 0 for the uniform and normal distribution. In a word, when *α* = 0, this algorithm has no effect. Therefore, the non-weighting approach is not suitable for the minimal test-cost feature subset problem.
2. The average *α* approach takes a statistical approach and significantly improves the quality of the results in each dataset. For the *Promoters*, *Prostate-GE*, *Credit-g*, and *SMK-CAN-187* datasets, the approach has especially good results. For the *SMK-CAN-187* dataset, the value increases by about 99.1% for the uniform distribution. Relatively good results are obtained for the other datasets. For example, for the uniform distribution, the best value of the *α* = 0 approach is 0.440, and the value of the average *α* approach is 0.979, an increase of 52.9%. This result is a big improvement.

### Effectiveness compare with two algorithms

In this section, we compare the proposed algorithm with two existing algorithms [43, 44] to show the efficiency of our algorithm. First, the two existing algorithms and the CSFS-RSLS algorithm are used in a support vector machine classifier to compute the classification accuracy. We used 60% of the dataset as the training set and the rest as the test set. Second, using the uniform distribution, each algorithm was run 100 times with different test cost settings and the optimal factor was compared with different exponential weight settings.

**Fig 28:**
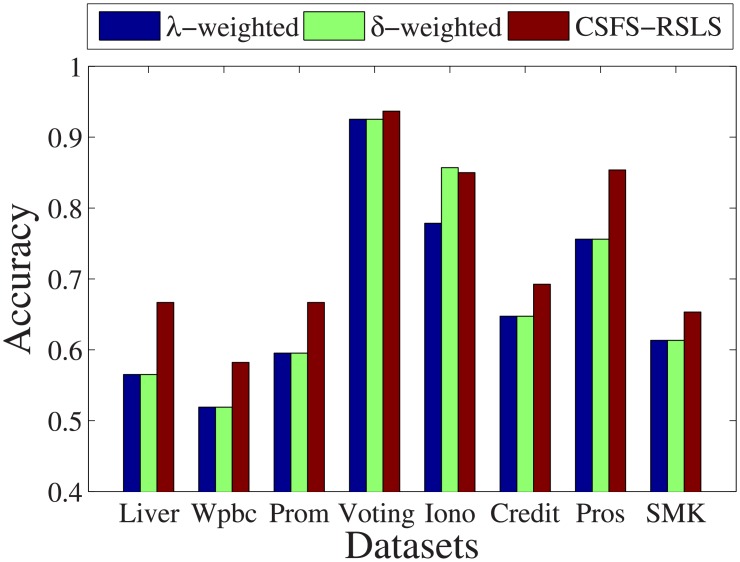
Classification accuracy.

Fig 28 shows the classification accuracy of the three algorithms for eight datasets. For the *Liver*, *Wpbc*, *Promoters*, *Voting*, *Credit*, *Prostate-GE*, and *SMK-CAN-187* datasets, the classification accuracy of the *λ*-weighted algorithm and *δ*-weighted algorithm is the same. For these datasets, the CSFS-RSLS algorithms has a higher classification accuracy than these two algorithms. Even for the *Prostate-GE* dataset, the classification accuracy of the CSFS-RSLS algorithm is higher than that of the two algorithms by about 10%. For the *Ionosphere* dataset, our CSFS-RSLS algorithm is only lower than the *δ*-weighted algorithm by about 1%. However, it is higher than the *λ*-weighted algorithm by about 7%.

**Fig 29:**
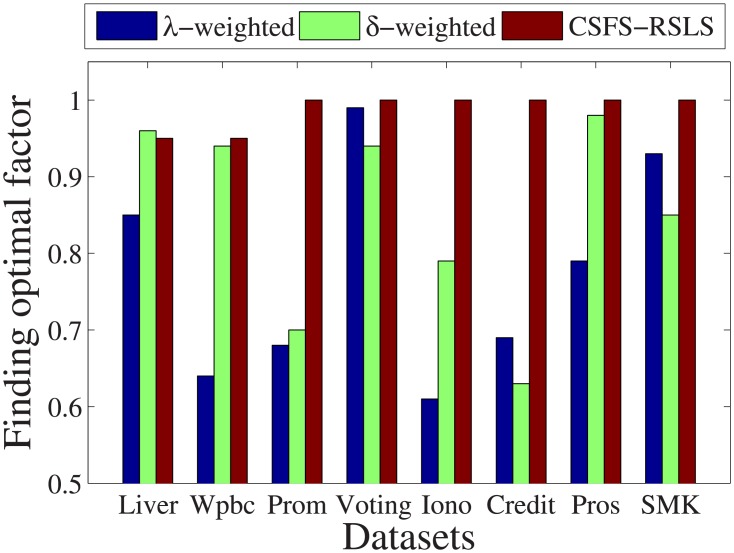
Finding optimal factor.

Fig 29 shows the optimal factor found by the three algorithms with the optimal exponential weight. For the *Promoters*, *Voting*, *Ionosphere*, *Credit*, *Prostate-GE*, and *SMK-CAN-187* datasets, the optimal factor found by the CSFS-RSLS algorithm is 1. For the *Ionosphere* dataset, the optimal factor found by the CSFS-RSLS algorithm is higher than that of the *λ*-weighted algorithm by about 0.4. This is an unsatisfactory number. For the *Liver* dataset, the optimal factor found by the CSFS-RSLS algorithm is lower than that of the *δ*-weighted algorithm by about 0.01. This value is acceptable.

## Conclusion and further work

In this paper, we have developed a new method for cost-sensitive feature selection. Firstly, we use rough sets to calculate the core of all features and use LS to calculate the importance of the each feature. Secondly, the cost is randomly generated by the three different distributions. Finally, we combine the feature importance and cost. To compare the performance of the proposed algorithm, we use two heuristic algorithms to our paper in the same experimental environment. Extensive experimental results show that the proposed algorithm can have better performance and obtain a feature subset with low cost. The CSFS-RSLS algorithm also outperforms the existing algorithms.

With regard to further work, many tasks need to be undertaken. First, other realistic data models with test costs can be built. A second point to be considered in future research is that the misclassification cost also should be added to the model. A model combining the test cost with the misclassification cost will be more suitable for real application. In the future, we will focus on designing more effective and efficient algorithms to cope with the minimal cost feature-selection problem. In summary, this study suggests new research trends for the feature selection problem and cost-sensitive learning.
